# Impact of Water Shortage on Soil and Plant Attributes in the Presence of Arbuscular Mycorrhizal Fungi from a Harsh Environment

**DOI:** 10.3390/microorganisms11051144

**Published:** 2023-04-28

**Authors:** Antonio Marcos Miranda Silva, Henrique Petry Feiler, Xue Qi, Victor Lucas Vieira Prudêncio de Araújo, Gileno Vieira Lacerda-Júnior, Paulo Ivan Fernandes-Júnior, Elke Jurandy Bran Nogueira Cardoso

**Affiliations:** 1 Soil Science Department, “Luiz de Queiroz” College of Agriculture, University of São Paulo, Piracicaba 13418-900, São Paulo, Brazil; 2 Department of Agronomy, Purdue University, West Lafayette, IN 47906, USA; 3 College of Grassland, Resources, and Environment, Inner Mongolia Agricultural University, Hohhot 010018, China; 4 Brazilian Agricultural Research Corporation—Embrapa, Jaguariúna 13918-110, São Paulo, Brazil; 5 Brazilian Agricultural Research Corporation—Embrapa Semiárido, Petrolina 56302-970, Pernambuco, Brazil

**Keywords:** water deficit, mycorrhizal association, fungal inoculation, water stress mitigation, corn

## Abstract

Arbuscular mycorrhizal fungi (AMF) play a crucial role in plant health due to their ability to improve tolerance to biotic and abiotic stresses. Our aim was to evaluate the effectiveness of a pool of native AMF from a harsh environment on plant performance and changes in soil attributes under different levels of drought. An experiment using maize was established, varying the soil water content to simulate severe drought (30% of the water-holding capacity [WHC]), moderate (50% of the WHC) and no drought (80% of the WHC, control treatment). Soil and plant attributes were measured (enzyme activity, microbial biomass, AMF root colonisation and plant biomass and nutrient uptake). There was a two-fold increase in plant biomass under moderate drought when compared to no drought treatment, but there was no difference in nutrient uptake. Under severe drought, there were the highest enzyme activities related to phosphorus (P) cycling and P microbial biomass, indicating higher P microbial immobilization. The increase in AMF root colonisation was observed in plants under moderate and no drought. Our findings demonstrated that the better use of the AMF inoculum varied according to drought levels, with better performance under moderate drought due to the increase in plant biomass.

## 1. Introduction

One of the main concerns in agriculture is the increasing frequency of drought events observed in recent decades. The foremost impact of drought is to increase soil water shortage and reduce nutrient mobility, thereby reducing crop yields, leading to severe economic losses and potential threats to food security [1]. Soil water shortage is further intensified in arid and semi-arid regions due to scarce rainfall [2], leading to production shortfalls in key crops such as maize (*Zea mays* L.), rice (*Oryza sativa* L.) and wheat (*Triticum aestivum*) [3].

For instance, in Brazil, the world’s second-largest maize exporter, losses of up to 41% in 2022/2023 crop yields were experienced due to the intensification of the drought, with projections indicating that the situation could worsen [3,4,5]. Considering the significance of maize as one of the most important food crops worldwide, it is imperative to develop eco-friendly strategies to ensure global food security and production, particularly in the context of climate change [6,7].

In the current scientific context of bio-economy and agriculture sustainable management, the use of arbuscular mycorrhizal fungi (AMF) has become a subject of growing interest. AMF, which belong to the monophyletic phylum Glomeromycota, form mutualistic associations with most vascular plants (more than 80%), making them ubiquitous [8,9]. The earliest evidence of AMF dates back over 450 million years and played a crucial role in facilitating the colonisation of land by plants, with both partners (the mycobiont: AMF, and the phytobiont: plants) coevolving over time [10,11]. The established intimate association of AMF with plant roots is known as mycorrhizal symbiosis, which is often associated with enhanced root growth, nutrient uptake, biotic and abiotic stress tolerance and improved crop and forest productivity [12,13,14,15,16].

Evidence to date shows that the use of AMF is capable of overcoming various types of stress, including drought [17,18], salinity [19] and heavy metals toxicity [20]. These beneficial effects arise from a complex interplay of physiological and biochemical signals exchanged between the mycobiont and phytobiont partners. Physiological responses include the activation of enzymatic antioxidant defence systems, such as superoxide dismutase (SOD), catalase (CAT) and ascorbate peroxidase (APX), as well as the synthesis of non-enzymatic antioxidants such as glutathione, cysteine, tachopherols and thioredoxin, which scavenge reactive oxygen species (ROS). Biochemical responses involve the regulation of hormones, such as abscisic acid, gibberellin and indole acetic acid, as well as the expression of strigolactone synthesis genes, up-regulation of antioxidant genes, down-regulation of polyamines catabolic enzyme genes and increase of cell membrane transporters. Additionally, morphological adaptability, including improvements in root architecture and hydraulic conductivity facilitated by the network of mycorrhizal hyphae, represents a significant mechanism of interaction between AMF and plant [17,18,19,20,21,22,23].

Despite the significant role of AMF in plant health, limited knowledge exists regarding the impact of using native AMF inoculum from tropical harsh environments on plant performance under different levels of drought stress. Therefore, our study was set up to test the hypothesis that the effectiveness of a native AMF inoculum, obtained from a harsh environment, would vary according to the severity of drought stress, leading to changes in soil and plant microbiological parameters related to nutrient cycling and the plant antioxidant system. Specifically, our aim was to evaluate the plant performance under severe, moderate, and no drought conditions, and soil attributes’ response when a native AMF inoculum was applied.

## 2. Materials and Methods

### 2.1. Experimental Design

The experiment was established in a one-factor completely randomized design with three levels and five replicates. The single factor was the drought levels (30%, 50% and 80% of the water-holding capacity (WHC), simulating severe drought, moderate drought and no drought, respectively).

Each experimental unit comprised a 3 L pot (20 cm inner diameter and 15 cm high), consisting of 1.7 kg of soil on a dry basis, thereby constituting a mesocosm experiment. The soil used for this experiment was obtained from the Ah horizon (0–10 cm deep) of a field site located in Brazil (22°42′ S, 47°38′ W) and classified as an Arenosol [24], with a texture of sandy clay loam (22.6% clay, 23.0% silt, 75.1% sand). According to the soil chemical characterisation [25], soil presented a pH (soil:CaCl_2_ ratio of 1:2.5) of 6.5, organic matter of 15 g kg^−1^, low available P content (<6 mg kg^−1^), extractable S of 18.0 mg kg^−1^ and cation-exchange capacity of 35.8 mmol_c_ kg^−1^.

Maize seeds (*Zea mays* L. (cv. BRS Gorotuba)) were surface sterilized and germinated in Petri dishes at 25 °C in the dark (obtaining 90% germination rate) prior to being planted in plastic pots. Two plants were maintained per experimental pot. Initially, pots had ^1^/_3_ of their capacity filled with the autoclaved soil (at 121 °C for 2 h). Then, 50 g of AMF inoculum was added (see Section 2.3) and the remaining volume of the pot was completed with the autoclaved soil.

### 2.2. Water-Holding Capacity Determination and Experimental Management

Soil WHC was monitored and weighed daily, and distilled water was added when necessary to reach the moisture content of interest. The soil WHC was determined based on the water retention curve (WRC). Briefly, samples were initially saturated by gradually raising a water layer. Then, they were weighed to estimate the water content at saturation (*θs*). Next, samples were taken to determine the water in equilibrium at potentials (*Ψ*) at −0.01, −0.04, −0.06 and −0.1 kPa on an automated tension table, and at −0.3, −0.5 and −1.0 kPa in Richards chambers. After reaching water balance at each potential, the samples were weighed. Subsequently, they were dried in an oven at 105 °C to quantify the water content (*θ*), in cm^3^ cm^−3^, associated with each *Ψ*.

**Figure A1:**
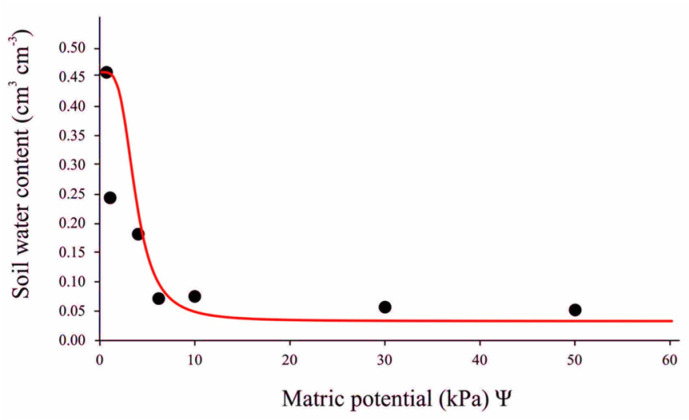
Water retention curve of soil used for the experiment.

The WRCs were modelled using van Genuchten’s model (Equation (1)) which associates the moisture values obtained at equilibrium with the respective *Ψ*, with Mualem’s restriction (Equation (2)) [26].

(1) $$\theta =\theta r+\frac{\left(\theta s-\theta r\right)}{{\left[1+{\left(\alpha \;\Psi \;m\right)}^{n}\right]}^{m}}$$

(2) $$m=1-\left(\frac{1}{n}\right)$$

where *θ* is the volumetric soil moisture (cm^3^ cm^−3^). *θr* is the residual volumetric soil moisture at 1500 kPa tension (cm^3^ cm^−3^). *θs* is the volumetric moisture of the saturated soil (cm^3^ cm^−3^). *Ψ* is the soil water potential (kPa). *α* and *n* are the empirical parameters of the equation, obtained by fitting the model, and *m* is the empirical parameter of the equation, obtained according to Mualem’s restriction. The parameters were obtained using the ‘soilphysics’ package [27] and the curve was plotted using the ‘ggplot 2’ package [28]—Appendix A (Figure A1).

Maize plants were cultivated following the same water management described by Silva et al. [16], where they were kept under severe drought (30% of WHC), moderate drought (50% of WHC) and no drought (80% of WHC, control treatment) for 120 days until the harvesting procedure. The average daily temperature and relative humidity in the greenhouse were 23 °C and 46%, respectively. Daily temperature and humidity during the experiment are provided in Supplementary Materials (Figure S1). The Hoagland solution was applied to keep the nutritional balance of the plants. The final solution of pH 5.5 was composed of 4 mM Ca(NO_3_)_2_, 6 mM KNO_3_, 2 mM MgSO_4_, 1 mM Fe-EDTA and 1 mM trace elements [29].

Notably, our experiment was specifically designed to investigate the role of the AMF inoculum against drought levels. Therefore, all plants were inoculated, since the aim of our investigation was to evaluate the soil and plant response in the presence of AMF inoculum obtained from a harsh environment, varying only the water soil content. Thus, the control treatment was considered the optimal water condition (no drought). Xiao et al. [30] have used this approach to assess the effect of drought stress on soil quality parameters in experiments with different plant species. Likewise, Sendek et al. [31] used this approach to explore the effects of plant genotype richness and AMF richness on plant yield under ambient and drought conditions. Still, Torres-Arias et al. [32] used a similar approach aiming to find the most appropriate abiotic conditions for the production of native AMF.

### 2.3. AMF Inoculum Characterization

The AMF inoculum consisted of the rhizosphere soil of *Tripogonella spicata* (Nees) plants, the so-called resurrection grass, due to their surprising rehydration capacity after a drought period [33,34].

Briefly, AMF inoculum were collected at the Brazilian Agricultural Research Corporation (Embrapa Semi-arid; 8°48′11.6″ S, 40°14′48.4″ W), in north-eastern Brazil, where a bio-prospecting program was developed to find microbes with the potential of helping crop plants to tolerate drought stress [16,35,36].

**Figure A2:**
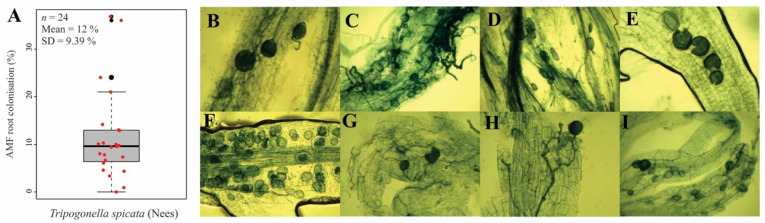
Root arbuscular mycorrhizal colonisation (AMF) traits. (**A**) boxplot showing all samples (*n* = 24) that composed the AMF inoculum (red dots) and outliers’ presence (black dots). SD: standard deviation. (**B**) AMF spores inside the root. (**C**) fungal mycelium. (**D**) AMF vesicles (oval shaped). (**E**,**F**) spores and fungal hyphae. (**G**–**I**) spores germinated inside the root.

**Figure A3:**
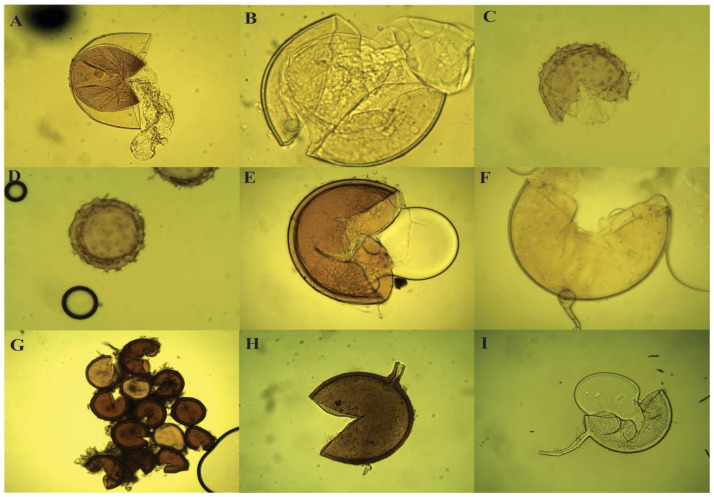
Arbuscular mycorrhizal fungi that composed the soil inoculum. (**A**) *Acaulospora morrowiae* (PVLG + Melzer) 20×. (**B**) *Acaulospora morrowiae* (PVLG) 40×. (**C**,**D**) *Ambispora* sp. (PVLG) 20×. (**E**) *Gigaspora decipiens* (PVLG + Melzer) 20×. (**F**) *Gigaspora gigantea* (PVLG) 10×. (**G**) *Glomus glomerulatum* (PVLG + Melzer) 10×. (**H**) *Glomus* sp. (PVLG + Melzer) 20×. (**I**) *Rhizophagus clarus* (PVLG) 10×.

The AMF inoculum consisted of soil containing mycorrhizal roots (with 12% of AMF root colonisation, Appendix B, Figure A2), spores and extraradical mycelium. On average, 620 AMF spores were composed of 50 g of AMF inoculum. These spores belong to the genera *Acaulospora*, *Ambispora*, *Gigaspora*, *Glomus* and *Rhizophagus* and a description of the species is provided in Table 1, while their morphological features are displayed in Appendix C (Figure A3).

### 2.4. Soil and Plant Sampling

Soil and plants were collected 120 days after planting, with soil stored in a 50 cm^3^ polypropylene tube at 4 °C for biological analysis or 25 °C for chemical analysis. Two fresh diagnostic leaves (newly expanded laminae leave) were collected and immediately wrapped in an aluminium bag, frozen in liquid N and stored at −80 °C prior to enzyme activities analysis.

### 2.5. Analytical Analyses

#### 2.5.1. Plant Biomass and Nutrient Content

Plant samples (shoot and root) were dried in a forced-air oven at 65 °C for 72 h to measure dry weight and then ground in a Wiley mill prior to the determination of nutrient content. Nutrient contents (N, P, K, Ca, Mg and S) were analysed by acid digestion, followed by steam distillation [37].

#### 2.5.2. Soil and Plant Enzyme Activities

In soil, inorganic pyrophosphatase (EC 3.6.1.1), acid (EC 3.1.3.2) and alkaline phosphatases (EC 3.1.3.1) activities were evaluated due to their direct role in phosphorus (P) cycling in the soil and were determined following the methodology proposed by Dick and Tabatabai [38] and Tabatabai [39]. Briefly, inorganic pyrophosphatase was determined using an extraction and colourimetric determination of the orthophosphate (Pi) released when 1 g soil was incubated with buffered (pH 8) pyrophosphate (PPi) solution at 37 °C for 5 h. The 1 N H_2_SO_4_ was used to extract Pi and the calorimetric method was used to determine the Pi extracted in the presence of PPi [38]. Whilst phosphatases were determined using incubation with a buffer solution (at pH 6.5 for acid phosphatase or at pH 11 for alkaline phosphatase) and a soil-buffer mixture of *p*-nitrophenyl-phosphate (PNF) [39].

In plants, ascorbate peroxidase APX (EC 1.11.1.11), catalase CAT (EC 1.11.1.6) and superoxide dismutase SOD (EC 1.15.1.1) activities were evaluated due to their role in the plant antioxidant system, i.e., responsible for the detoxification of reactive oxygen species (ROS). First, plant material was macerated in the presence of liquid nitrogen prior to extraction buffer addition according to the extraction procedure of Azevedo et al. [40]. The extract was used to determine: (i) APX activity according to Moldes et al. [41] in a spectrophotometer at 290 nm. (ii) CAT activity according to Kraus et al. [42] modified by Azevedo et al. [40] in a spectrophotometer at 240 nm. (iii) SOD activity according to Ciannopolitis and Ries [43] and Cembrowska-Lech et al. [44] in a spectrophotometer at 560 nm.

#### 2.5.3. Soil Microbial Biomass (Carbon and Phosphorus)

Microbial biomass carbon (MBC) and phosphorus (MBP) were extracted using the fumigation-extraction method proposed by Brookes et al. [45,46]. MBC was determined by titration according to Vance et al. [47], whilst MBP was determined by spectrophotometry (882 nm) according to Murphy and Riley [48].

#### 2.5.4. Soil Glomalin and AMF Root Colonisation

Glomalin, a glycoprotein synthesized majority by arbuscular mycorrhizal fungi, was obtained from autoclave extraction (at 121 °C for 30 min), using 1 g of soil and 8 mL of 20 mM sodium citrate solution (pH 7.4). Then, samples were centrifuged at 5000× *g* for 20 min and the supernatant was removed for quantification according to the method proposed by Bradford [49] and Wright and Upadhyaya [50].

For mycorrhizal root colonisation, roots (<2 mm in diameter) were washed with tap water and immersed in 10% potassium hydroxide (KOH) for 24 h at room temperature (25 °C). Then, the tubes with root segments were transferred to a 90 °C water bath for 30 min with the addition of 10% hydrogen peroxide (H_2_O_2_) to completely bleach the segments [51]. The roots were washed in tap water and then received a staining solution with blue ink (PakerQuink^®^, Parker, Nantes, France) and 5% acetic acid and finally, they were immersed in a 90 °C water bath for 15 s [52]. The roots were washed in tap water and were preserved in a lactoglycerol solution (1:1:1, lactic acid, glycerol and water). The evaluation of the percentage of mycorrhizal colonisation was performed using the intersect method for a minimum of 100 intersects [53] using a stereoscopic microscope (Leica MZ12.5, Leica Microsystems, Wetzlar, Germany). AMF colonisation represents the proportion (percentage) of the root occupied by any AMF structure (arbuscules, hyphae, vesicles or spores.

### 2.6. Data Analyses

As a univariate approach, we used the one-way analysis of variance (ANOVA) to identify significant differences between drought levels in plant dry biomass, nutrient uptake, soil and plant enzyme activities, soil microbiological parameters (microbial biomass carbon and phosphorus and glomalin content) and AMF root colonisation. Previously, data were tested for normal distribution using the Shapiro–Wilk test, followed by the homogeneity of variances tests, using the Bartlett test. Tukey’s test was used as a posthoc pairwise comparison (*p* < 0.05) using the ‘ExpDes’ package [54]. We used the ‘ggplot2’ package [28] to create the stacked bar graph and boxplot graphs.

Principal component analysis (PCA) was performed as a multivariate approach to integrate all obtained data, correlating variables with the drought levels. Firstly, data were transformed into a log (*x* + 1) to meet the multivariate normality, and the attributes subject to collinearity were removed [55]. For the PCA, we used the statistical packages ‘FactoMineR’ [56] and ‘factoextra’ [57]. All statistical analyses were performed in the R^®^ program [58].

## 3. Results

**Figure 1:**
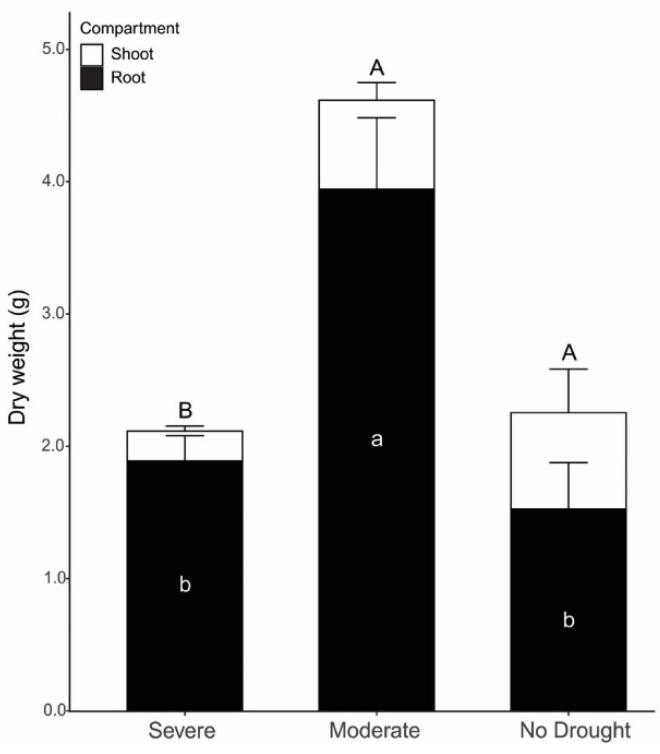
Plant dry weight under severe, moderate and no drought conditions, considering root (black bar) and shoot (white bar) compartments. Uppercase letters compare differences in shoot compartment, while lowercase letters compare differences in root compartment by Tukey’s test at 5% (*p* < 0.05). Standard deviation is shown (*n* = 5).

Dry biomass results revealed increases of 2.1 and 2.6-fold in root dry weight (RDW) under moderate drought (3.94 ± 0.49 g) when compared to severe drought (1.89 ± 0.19 g) and no drought conditions (1.53 ± 0.32 g), respectively. Shoot dry weight (SDW) did not differ between moderate drought (0.67 ± 0.06 g) and no drought (0.73 ± 0.15 g) and differed only from severe drought (0.23 ± 0.02 g). Therefore, under moderate drought, a 2-fold increase in the total plant dry weight was observed (4.61 g) when compared to no drought treatment (2.26 g) (Figure 1 and Figure S2).

There was no difference (*p* > 0.05) in nutrient uptake between treatments and, overall, N e P was allocated more in RDW than in SDW. There was no clear allocation pattern for the other nutrients (Table 2).

**Figure 2:**
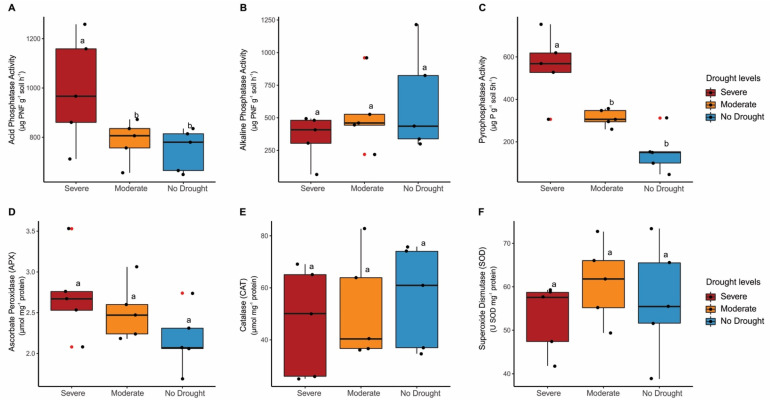
Response of soil (**A**–**C**) and plant (**D**,**E**) enzyme activities under severe, moderate and no drought conditions. (**A**) acid phosphatase activity, (**B**) alkaline phosphatase activity, (**C**) inorganic pyrophosphatase activity, (**D**) ascorbate peroxidase (APX) activity, (**E**) catalase (CAT) activity and (**F**) superoxide dismutase (SOD) activity. Boxplot displays the minimum, first quartile, median (vertical line), third quartile and maximum, respectively. Black dots indicate replicates (*n* = 5), while red dots indicate the presence of outlier. Lowercase letters compare differences between drought levels by Tukey’s test at 5% (*p* < 0.05).

In soil, acid phosphatase and inorganic pyrophosphatase activities differed (*p* < 0.05) according to drought levels, with severe drought showing the highest activities (991 µg PNF g^−1^ soil h^−1^ and 555 µg P g^−1^ soil 5 h^−1^, respectively). Still, the lowest activities were observed under moderate drought (785 µg PNF g^−1^ soil h^−1^ and 313 µg P g^−1^ soil 5 h^−1^) and no drought (749 µg PNF g^−1^ soil h^−1^ and 153 µg P g^−1^ soil 5 h^−1^) and did not differ (*p* > 0.05) from each other. Alkaline phosphatase activity remained constant between drought levels (Figure 2A–C). Therefore, drought levels only affected acid phosphatase and inorganic pyrophosphatase activities in soil, with severe drought showing the highest activities.

In plants, no difference (*p* > 0.05) was observed in the enzyme activities related to the antioxidant defence system (APX, CAT and SOD). On average, APX tended to decrease with increasing soil water content, ranging from 2.7 µmol mg^−1^ protein (severe drought) to 2.1 µmol mg^−1^ protein (no drought). On the other hand, CAT tended to increase with increasing soil water content, ranging from 47 µmol mg^−1^ protein (severe drought) to 56 µmol mg^−1^ protein (no drought). For SOD, activity of 53 U SOD mg^−1^ protein for plants under severe drought was observed, 61 U SOD mg^−1^ protein for plants under moderate drought and 57 U SOD mg^−1^ protein for plants under no drought (Figure 2D–F). Overall, despite the average trend towards depletion and increased activities of antioxidant enzymes, no significant difference was observed between levels of drought.

**Figure 3:**
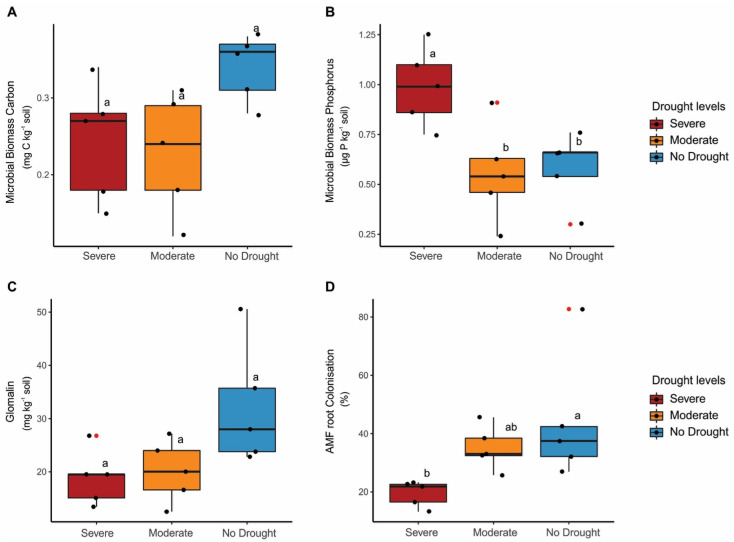
Response of (**A**) microbial biomass carbon, (**B**) microbial biomass phosphorus, (**C**) glomalin content and (**D**) arbuscular mycorrhizal fungi (AMF) root colonisation under severe, moderate and no drought conditions. Boxplot displays the minimum, first quartile, median (vertical line), third quartile and maximum, respectively. Black dots indicate replicates (*n* = 5), while red dots indicate the presence of outlier. Lowercase letters compare differences between drought levels by Tukey’s test at 5% (*p* < 0.05).

Although microbial biomass carbon (MBC) and glomalin content did not differ (*p* > 0.05) between treatments, they appeared to be higher in the moderate and no drought treatments. Whilst microbial biomass phosphorus (MBP) and AMF root colonisation showed differences (*p* < 0.05) between treatments but opposite behaviour. The highest AMF root colonisation was found in plants under no drought, not differing from plants under moderate drought. Whilst the lowest AMF root colonisation was found in plants under severe drought (Figure 3A–D). In summary, the highest MBP was found under severe drought, while the highest AMF root colonisation was found under no drought and moderate drought.

**Figure 4:**
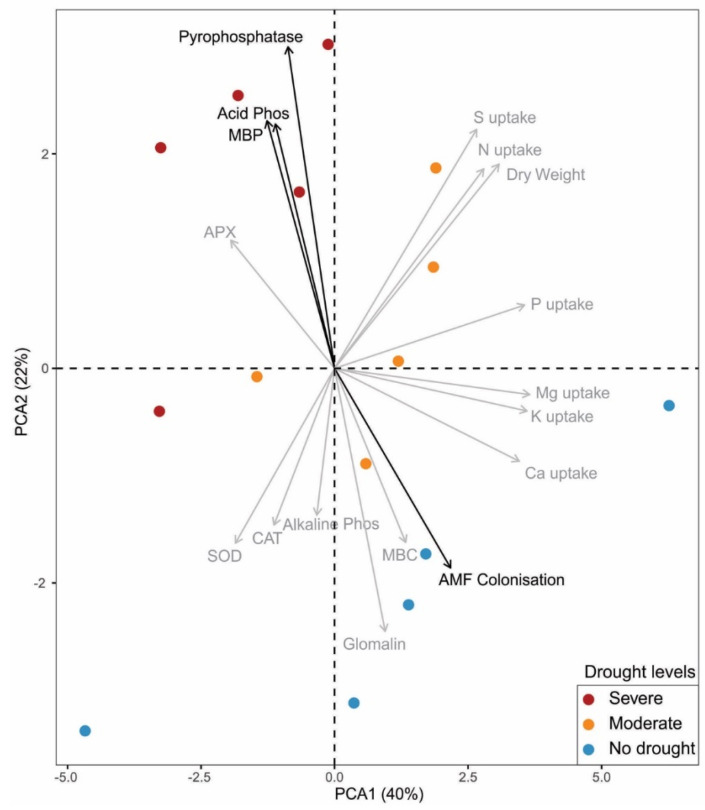
Principal component analysis (PCA) displaying relationships between the variables evaluated and treatments under severe, moderate and no drought conditions. Black arrows indicate significance in one-way ANOVA analysis, while grey arrows indicate non-significance. Pyrophosphatase: inorganic pyrophosphatase activity; Acid Phos: acid phosphatase activity; MBP: microbial biomass phosphorus; APX: ascorbate peroxidase activity; SOD: superoxide dismutase activity; CAT: catalase activity; Alkaline Phos: alkaline phosphatase activity; MBC: microbial biomass carbon; AMF colonisation: arbuscular mycorrhizal fungi root colonisation; Dry weight: total plant dry weight.

According to the principal component analysis (PCA), the first two components explained 62% (PCA1 = 40% and PCA2 = 22%) of the data variation, ensuring a better interpretation of the results (Figure 4). Data dispersion between replicates was lower under severe and moderate drought compared to no drought samples. MBP, APX, acid phosphatase and pyrophosphatase activities were highly correlated with each other and more associated with the severe drought. Whilst MBC, glomalin content, AMF root colonisation, Mg, K and Ca uptake were more associated with the no drought condition. The total plant dry weight and nutrient uptake (N, P and S) correlated with each other and were associated with plants under moderate drought. The PCA results corroborate and complement the results seen in the aforementioned one-way ANOVA. In summary, drought levels clustered differently in the PCA, with severe drought being more correlated with soil parameters associated with phosphorus cycling, while no drought showed a correlation with AMF root colonisation.

## 4. Discussion

Our findings demonstrated that the use of the AMF inoculum from a harsh environment was able to increase maize biomass and change the response of soil attributes, especially under moderate drought. We partially accepted our initial hypotheses, since the effectiveness of the native AMF inoculum varied according to drought levels, but did not promote significant changes in the plant’s antioxidant system. This general understanding is vital for planning the better use of AMF inoculum not only to increase crop resilience to drought, but also to maximize crop yields, especially in the context of rising drought events. However, it is crucial to consider that, in general, mesocosm experiments hamper plant potential growth due to various factors, such as the limitation of pot size and nutrient cycling exchanges. This constraint may be intensified by the depletion of the soil microbiota brought about by the process of soil autoclaving. As a result, the small dry weight observed in our investigation is consistent with the previous finding by Rubin and Görres [59].

According to Xiao et al. [30] moderate drought stress exerts a positive effect on soil quality in the grass’s species due to the increased fine root turnover and rhizodeposition. Likewise, previous studies reported that moderate drought stress facilitated the growth of fine roots, leading to an increase in the root-to-shoot biomass ratio [60,61]. Likewise, in our investigation, we observed a significant increase in root biomass under moderate drought. This result may be associated with the intrinsic characteristic of the AMF inoculum, since it was obtained from the rhizosphere of the plant known as the resurrection plant (*Tripogonella spicata*). *T. spicata* plants can survive extreme dehydration, undergoing a reversible transition from a desiccated, metabolically inactive state to a hydrated, metabolically active state without suffering any permanent damage [22]. These plants harbour a unique microbial community which, combined with their physiological apparatus, gives them a drought-tolerance capacity [33,34,35,36].

According to Torres-Arias et al. [32], investigating different abiotic factors for AMF inoculum production, the native AMF species were well adapted to the edaphoclimatic conditions of the area that were screened and, therefore, ensuring the success of the symbiosis and better development of the plants. This is leading different research groups to postulate the choice of native AMF inoculant instead of commercial or AMF isolates [62,63]. Therefore, local adaptation can represent a powerful factor in establishing new combinations of fungi and plants [64]. Indeed, according to the recent meta-analysis of Liu et al. [65], the use of microbial consortium increased plant growth by 48%, whilst single inoculation increased only 29%. Furthermore, the interaction between AMF and other microbes such as bacteria has been shown to increase nutrient uptake by plants and alleviate drought [16,66,67,68].

Overall, under drought, the soil microbial activity used to decline, including decreasing in enzyme production (e.g., phosphatases, pyrophosphatase) and nutrient cycling, leading to a decrease in soil fertility followed by lower plant productivity and loss in the economy [69]. This is also supported by Hosseini et al. [70], who found that severe drought considerably decreased the phosphatase activity by 67% in comparison with optimum moisture. On the other hand, our findings revealed an increase in soil microbial activity based on increases in acid phosphatase (up to 32%) and pyrophosphatase activities (up to 263%) in the soil under severe drought compared to no drought. These results suggest a greater demand for P under severe drought, which was not converted into biomass, since no increase in plant weight was observed. Meanwhile, a greater amount of microbial biomass phosphorus (MBP) was observed under severe drought, indicating that under severe drought events in the soil, the increase in the enzyme activities related to P availability was associated with P allocation in the soil microbial biomass, i.e., a greater P microbial immobilization. This result corroborates Sardans and Peñuelas [71] who found a decrease in phosphorus availability with increasing drought.

Although P immobilization is sensitive to drought, due to decreased diffusion and, subsequently, microbial uptake [72,73], our findings suggest that P immobilization was facilitated under severe drought. However, it is worth noting that microbial P immobilization may be temporary, and P may be released during microbial biomass turnover in response to moisture conditions or when carbon becomes limiting, for example [74].

In our investigation, increases in AMF colonisation, soil microbial carbon and glomalin content were observed in plants under no drought. According to de Vries et al. [75], plants under no water stress are actively growing and allocating photosynthate carbon to AMF in the rhizosphere, allowing different AMF taxa to colonize plants and, therefore, a higher AMF root colonisation is expected. On the other hand, plants under moderate drought have their photosynthesis and root exudation down-regulated, beneficing only direct interactions with AMF and bacteria, as well as reducing the heterotrophic microbial activity [75]. Here, these direct interactions would be linked to the increase in plant biomass observed under moderate drought (up to 104%) compared to no drought. According to Chareesri et al. [76], AMFs under drought stress are able to increase the plant hormone-driven pathways, conferring drought tolerance and high yields.

Here, no difference in plant enzyme activity related to the antioxidant defence system was observed and can be understood as alleviation of oxidative stress in plants due to AMF inoculum [77]. On the other hand, if considering the tendency observed, the better effect of the AMF inoculum could be more related to the decrease in ascorbate peroxidase (APX) activity or increase of superoxide dismutase (SOD) activities as observed in plants under the moderate drought that stood out in the growth of plant biomass.

Likewise, Amer et al. [78] showed that especially APX and catalase activities significantly decreased after AMF inoculation when water deficit was applied. These authors explained that this probably occurred due to the increased absorption of water provided by AMF hyphae and its transfer to the host plant, decreasing the generation of reactive oxygen species. However, Chandrasekaran [79] argues that the benefits of AMF inoculation to alleviate drought stress are more related to the reduction of non-enzyme antioxidants such as hydrogen peroxide, malondialdehyde, and electrolyte leakage.

## 5. Conclusions

Altogether, our study underscores the potential of AMF inoculum from the rhizosphere of a plant living in a harsh environment. We conclude that the native AMF inoculum effectiveness obtained from the rhizosphere of the resurrection plant *Tripogonella spicata* varies with soil water content based on biomass growth, but we provide no evidence that AMF inoculation could enhance nutrient uptake in maize plants. Furthermore, we conclude that changes in soil parameters related to phosphorus cycling (microbial biomass phosphorus, acid phosphatase and pyrophosphatase activity) and no change in the plant response of the antioxidant system may be related to a legacy effect of AMF inoculation. However, we argue that a better understanding would be achieved in a further investigation that addresses the AMF inoculum effectiveness considering its origin and soil water content, crops with different rhizodeposition patterns and genotypes and wide edaphoclimatic conditions.

## Figures and Tables

**Table 1:** Species composition of the AMF inoculum (50 g) used in this experiment.

AMF Species	Number of Spores/50 g of Inoculum
*Acaulospora morrowiae* Spain and N.C. Schenck	94
*Ambispora* sp.	417
*Gigaspora decipiens* I.R. Hall and L.K. Abbott	63
*Gigaspora gigantea* (T.H. Nicholson and Gerd) Gerd. and Trappe	9
*Glomus glomerulatum* Sieverding	7
*Glomus* sp.	9
*Rhizophagus clarus* (T.H. Nicholson and N.C. Schenck) C. Walker and A. Schüßler)	21
Total	620

**Table 2:** Nutrient uptake by maize, considering shoot, root and total dry weight under severe, moderate and no drought conditions.

*Compartment*/Drought Levels	N	P	K	Ca	Mg	S
*Shoot*	mg SDW^−1^					
Severe	3.80 ± 1.90 a *	0.22 ± 0.07 a	7.16 ± 1.97 a	1.08 ± 0.35 a	1.13 ± 0.42 a	0.43 ± 0.13 a
Moderate	8.58 ± 4.00 a	0.61 ± 0.40 a	23.13 ± 11.65 a	3.24 ± 1.63 a	3.21 ± 1.75 a	1.09 ± 0.54 a
No Drought	7.45 ± 5.31 a	0.74 ± 0.75 a	21.43 ± 17.00 a	3.12 ± 2.86 a	3.33 ± 3.61 a	1.23 ± 1.26 a
*Root*	mg RDW^−1^					
Severe	12.10 ± 6.02 a	1.43 ± 0.74 a	6.70 ± 3.26 a	1.68 ± 0.90 a	0.89 ± 0.48 a	5.22 ± 2.66 a
Moderate	15.47 ± 9.78 a	2.61 ± 1.61 a	7.54 ± 6.28 a	1.96 ± 1.30 a	0.93 ± 0.71 a	8.25 ± 5.48 a
No Drought	10.01 ± 10.22 a	2.97 ± 2.69 a	21.03 ± 17.04 a	12.34 ± 11.51 a	3.03 ± 2.74 a	3.79 ± 3.05 a
*Total dry weight*	mg plant^−1^					
Severe	15.90 ± 6.66 a	1.65 ± 0.75 a	13.86 ± 3.22 a	2.76 ± 0.99 a	2.02 ± 0.71 a	5.65 ± 2.65 a
Moderate	24.05 ± 9.45 a	3.22 ± 1.57 a	30.67 ± 9.45 a	5.20 ± 1.67 a	4.14 ± 1.54 a	9.34 ± 5.36 a
No Drought	17.46 ± 13.86 a	3.71 ± 3.33 a	42.46 ± 30.63 a	15.46 ± 13.78 a	6.36 ± 6.13 a	5.02 ± 4.08 a

* Lowercase letters compare differences between drought levels within the same compartment (shoot, root or total dry weight) by Tukey’s test at 5% (*p* < 0.05). Means are followed by standard deviation (*n* = 5).

## Data Availability

The data presented in this study are available on request from the corresponding author.

## Supplementary Materials

The following supporting information can be downloaded at: https://www.mdpi.com/article/10.3390/microorganisms11051144/s1, Figure S1: Daily average temperature and relative humidity in greenhouse from June 26 to September 26; Figure S2: Mesocosm experiment using a pool of arbuscular mycorrhizal fungi under varying drought levels. On the left side, the pot with 30% of the water-holding capacity (WHC) simulates severe drought, in the middle (50% of the WHC) pot simulates moderate drought, and on the right side the control treatment under no drought (80% of the WHC).
