# Supplementary material for: Impact of Water Shortage on Soil and Plant Attributes in the Presence of Arbuscular Mycorrhizal Fungi from a Harsh Environment
Source: Microorganisms. 2023 Apr 28;11(5):1144. doi: 10.3390/microorganisms11051144 (PMC10223447; doi:10.3390/microorganisms11051144)
Supplement: Supplementary file 1 [file microorganisms-11-01144-s001.zip › microorganisms-2333839-supplementary.pdf]

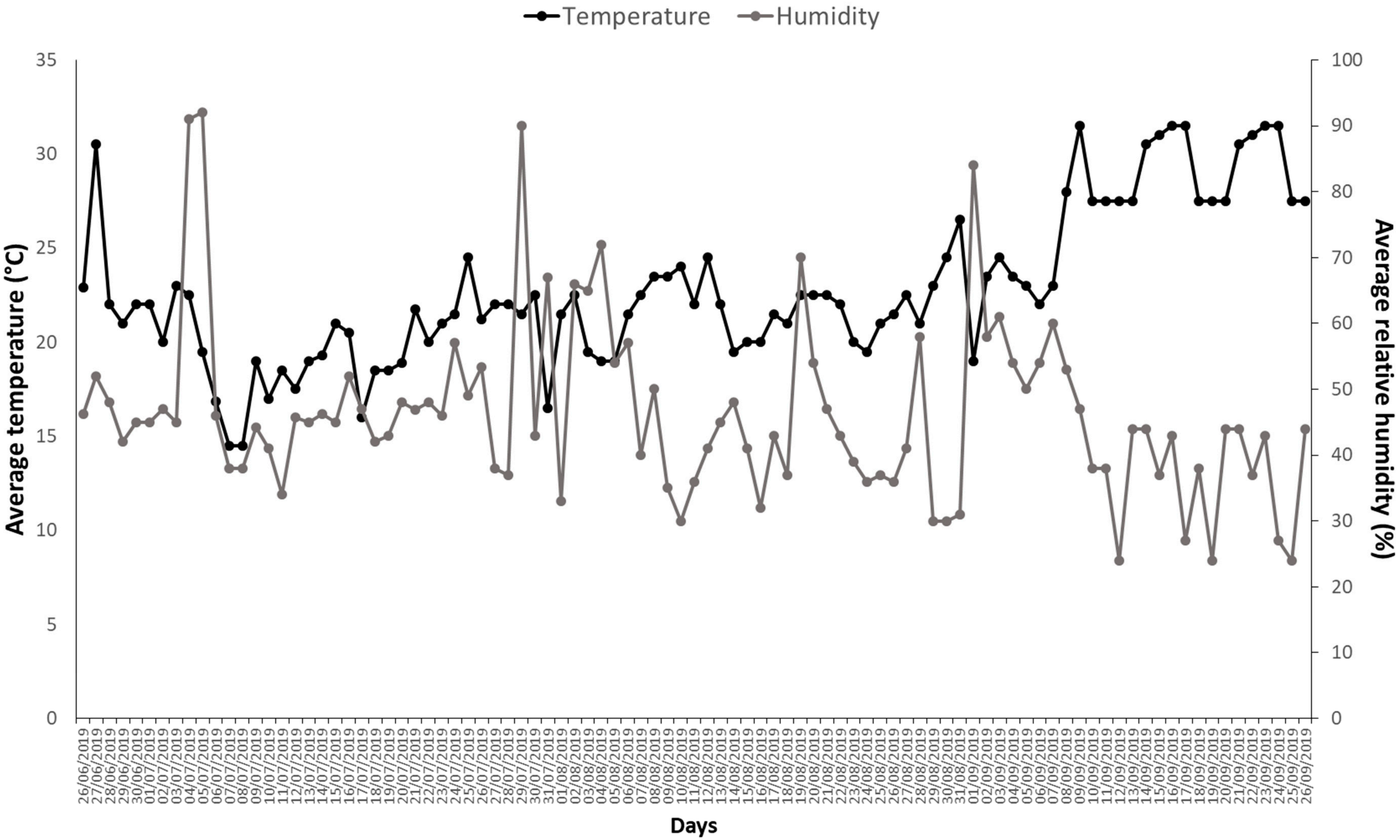

Figure S1. Daily average temperature and relative humidity in greenhouse from June 26 to September 26.

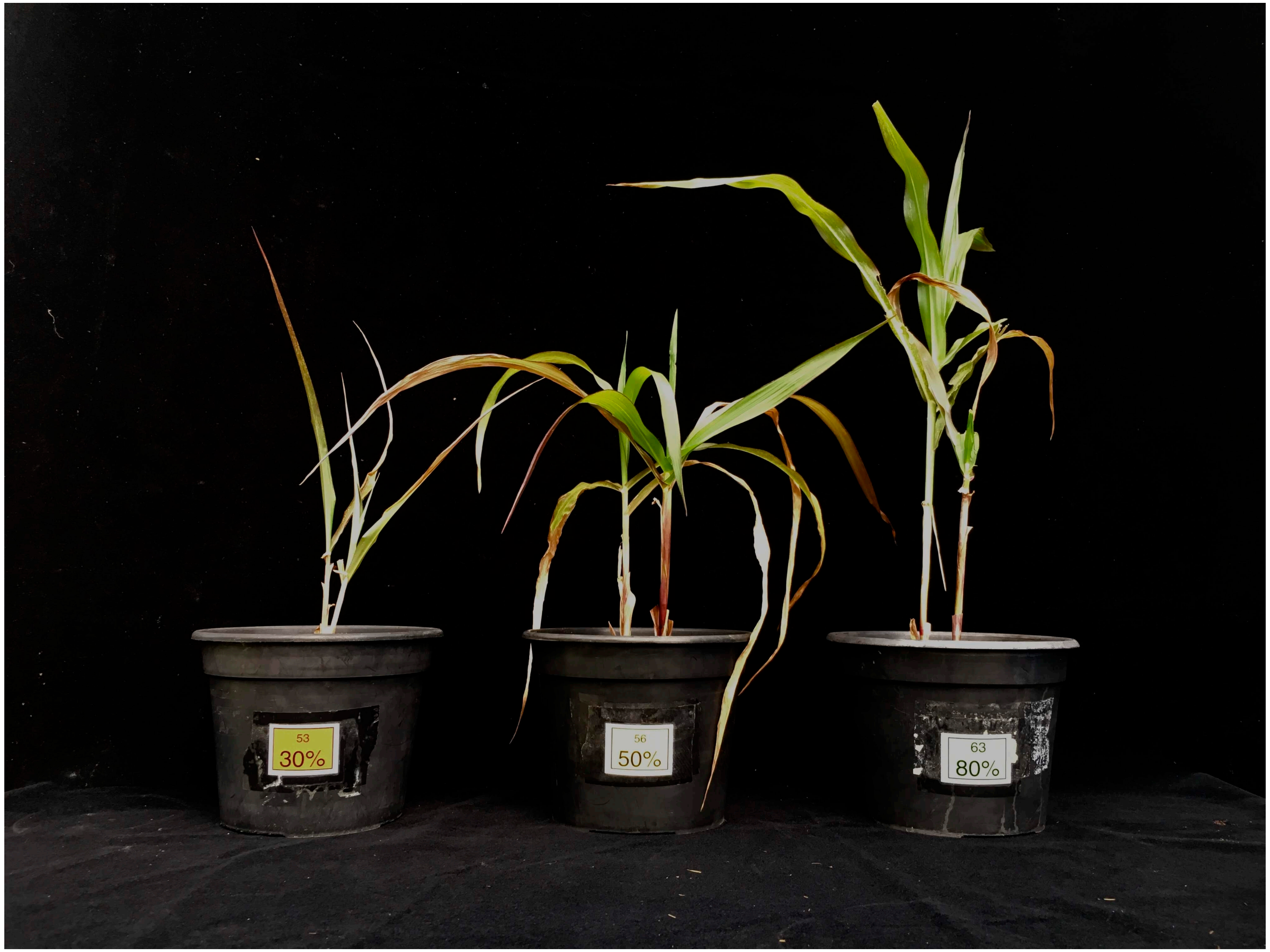

Figure S2. Mesocosm experiment using a pool of arbuscular mycorrhizal fungi under varying drought levels. On the left side, the pot with 30 % of the water-holding capacity (WHC) simulates severe drought, in the middle (50 % of the WHC) pot simulates moderate drought, and on the right side the control treatment under no drought (80 % of the WHC).
